# The Clinical Impact of Systematic Screening for Obstructive Sleep Apnea in a Type 2 Diabetes Population—Adherence to the Screening-Diagnostic Process and the Acceptance and Adherence to the CPAP Therapy Compared to Regular Sleep Clinic Patients

**DOI:** 10.3389/fendo.2018.00714

**Published:** 2018-11-29

**Authors:** Katerina Westlake, Veronika Dostalova, Andrea Plihalova, Martin Pretl, Jan Polak

**Affiliations:** ^1^Second Internal Medicine Department, Vinohrady Teaching Hospital, Prague, Czechia; ^2^Diabetology Practice Diabetologie Praha, Prague, Czechia; ^3^Department of Pathophysiology, Third Faculty of Medicine, Charles University, Prague, Czechia; ^4^Neurology and Sleep Laboratory, INSPAMED Ltd., Prague, Czechia; ^5^Institute of Sleep Medicine, Prague, Czechia; ^6^Diamant Neuropsychology Laboratory, Department of Neurology and Centre of Clinical Neuroscience, First Faculty of Medicine and General University Hospital in Prague, Charles University, Prague, Czechia

**Keywords:** sleep apnea, diabetes, screening, CPAP acceptance, CPAP adherence

## Abstract

Obstructive sleep apnea (OSA) is a common disorder in Type 2 diabetes (T2D) patients further increasing their already high cardiovascular risk. As T2D patients typically not report OSA symptoms, systematic screening for OSA in this population is warranted. We aimed to determine the readiness of T2D patients to undergo screening and to compare their adherence to continuous positive airway pressure (CPAP) therapy with “regular” sleep clinic patients who typically seek medical advice on their own initiative. We therefore recruited 494 consecutive T2D patients and offered them OSA screening using home sleep monitoring (type IV device). All participants in high risk of moderate-to-severe OSA were recommended home sleep apnea testing (HSAT) followed by CPAP therapy. Patients were followed-up for 12 months and outcomes compared to 228 consecutive sleep clinic patients undergoing HSAT. Among 307 screened T2D patients, 94 (31%) were identified at high risk of moderate-to-severe OSA. Subsequently, 54 patients underwent HSAT, 51 were recommended, and 38 patients initiated CPAP (acceptance 75%). Among 228 sleep clinic patients, 92 (40%) were recommended and 74 patients initiated CPAP (acceptance 80%). After 1 year, 15 (39%) T2D and 29 (39%) sleep clinic patients showed good CPAP adherence (use ≥ 4 h/night ≥ 70% nights). In conclusion, 20 T2D patients needed to be screened in order to obtain one successfully treated patient. OSA screening in T2D patients identified 31% with moderate-to-severe OSA. Once diagnosed, their CPAP acceptance and adherence did not differ from sleep clinic patients. However, the reasons for the high dropout during the screening-diagnostic process impacting the overall success of the screening program need to be identified and addressed.

## Introduction

Obstructive sleep apnea (OSA) is a common treatable disorder known to increase cardiovascular mortality (1–3). Even though the consequences of untreated OSA are severe, the majority of patients (~90%) with sleep apnea are not aware of their condition (4). Unawareness of OSA is concerning in the general population, where OSA affects 5–10% of people and even more so in a Type 2 diabetes population where the patients already are in a high risk of cardiovascular mortality due to diabetes and where the prevalence of OSA is particularly high reaching 60–80% (5–8). A number of studies also demonstrated an association between OSA, insulin resistance and impaired glucose tolerance showing that treatment of OSA may improve insulin sensitivity in patients with diabetes and pre-diabetes (9–11) although improvement of glucose control was not proved by randomized study in relatively well-controlled Type 2 diabetes patients (12). Furthermore, OSA is independently associated with diabetes-related microvascular complications-diabetic retinopathy and peripheral neuropathy which makes recognition and timely treatment of OSA in this population even more appealing (13, 14).

Given the impact of unrecognized and therefore untreated OSA in Type 2 diabetes patients, the International Diabetes Federation recommends screening Type 2 diabetes patients for OSA (15). As the screening questionnaires were shown to be inaccurate, having both low sensitivity and specificity, home sleep monitoring was suggested as an optimal tool to screen for OSA in a population of Type 2 diabetes patients (7, 16). Previously, we observed that OSA screening identified ~30% of Type 2 diabetes patients in high risk of moderate-to-severe OSA which confirms previously published data (5–8). These patients subsequently need to be diagnosed by a home or laboratory-based sleep study and referred to CPAP treatment when appropriate. As the major goal of systematic OSA screening is to ultimately improve quality of life and reduce morbidity and mortality, adherence to the whole process including screening, diagnosis and CPAP therapy represents a key factor determining its overall success.

Even though the consequences of untreated OSA and health benefits of CPAP are well-known and are easy to explain to patients, sleeping with a CPAP mask might present the patient with both psychological and physical discomfort. CPAP treatment requires a high degree of patient cooperation which becomes even more pronounced in screening-eligible Type 2 diabetes patients who were hitherto unaware of an additional health problem or did not consider OSA symptoms to be significant enough to, seek medical help and, in this case, were simply asked to follow their doctor's advice. Low CPAP acceptance and adherence is an ongoing challenge despite efforts to improve patient comfort and support, ranging from 25 to 73% (17–23) even in a sleep clinic population where the treatment usually comes as a follow up to a patient reported health issue. Data on patients approached through systematic screening are very limited (24, 25). So far, the adherence of patients with Type 2 diabetes to CPAP was typically observed if they also happened to be part of a sleep clinic population. Their attitudes to OSA screening, diagnosis and CPAP treatment when screened for OSA solely as a result of their doctor's initiative remain only partially elucidated (25).

In this study we hypothesized that Type 2 diabetes patients will show markedly lower acceptance and adherence to CPAP therapy as the OSA diagnosis resulted from a systematic screening performed in subjects who did not seek health care specialist for OSA related symptoms, in contrast to sleep clinic patients, who started CPAP treatment because they actively approached sleep physician. Additionally, we aimed to provide real-life data on the suitability, effectiveness and outcomes of systematic OSA screening in Type 2 diabetes patients by determining the adherence of patients with Type 2 diabetes to the systematic screening-diagnosis-therapy process in an outpatient clinics.

## Materials and methods

### Study of type 2 diabetes patients

#### Subjects

Subjects were recruited in diabetes care outpatient clinics providing routine care to unselected patients with diabetes located in Prague, Czech Republic between March 2014 and March 2015. In total, 494 consecutive patients fulfilled the inclusion criteria–diagnosis of Type 2 diabetes mellitus and age 18–80 years. Eleven patients were subsequently excluded due to unstable psychiatric disorders (5 patients) or already diagnosed OSA (6 patients) resulting in 483 subjects included in the study.

#### Study protocol—OSA screening, diagnosis and therapy

During regular scheduled visits (regular diabetes care), a physician educated all the subjects about OSA and the health risks associated with OSA as well as about the treatment options. At the same time, OSA screening by home sleep monitoring was offered in the form of a type IV device (ApneaLink, ResMed, San Diego, CA, United States) that recorded hemoglobin saturation, heart rate and nasal airflow during sleep. The respiratory event index (REI) was determined and subjects were stratified into low or high risk (REI ≥ 15) for the presence of moderate-to-severe OSA. Patients in the high-risk group were referred to a sleep clinic for a diagnostic home sleep study using a portable sleep monitor that recorded hemoglobin saturation, heart rate, nasal airflow, chest and abdominal respiratory effort and ECG (Nox T3, Nox Medical, Reykjavik, Iceland). Subsequently, patients with confirmed moderate-to-severe OSA were offered CPAP treatment according to AASM guidelines in an outpatient sleep center (Inspamed Prague, Czechia).

### Study of sleep clinic patients

#### Subjects and study protocol

The collaborating outpatient sleep center (Inspamed, Prague, Czechia) retrospectively included 252 consecutive patients who underwent diagnostic home sleep study in 2014 and were 18–80 years old. Subsequently, 24 patients were excluded for previous experience with OSA diagnosis or CPAP therapy, leading to 228 patients being evaluated in the study. Patients with moderate-to-severe OSA defined as REI ≥ 15 (none of them treated for diabetes) were offered CPAP treatment according to AASM guidelines. This study was carried out in accordance with the recommendations of the Ethical Committee of the Third Faculty of Medicine, Charles University, Prague with written informed consent from all subjects. All subjects gave written informed consent in accordance with the Declaration of Helsinki. The protocol was approved by the Ethical Committee of the Third Faculty of Medicine, Charles University, Prague.

#### Determination of acceptance and adherence

CPAP usage data of both, Type 2 diabetes and sleep clinic patients were analyzed at 3 and 12 months after establishing optimal mask fit, treatment pressure and regime (titration). Acceptance was defined as the patient's agreement to CPAP therapy after titration. Adherence was assessed using reports downloaded from CPAP machines. Patients using CPAP ≥4 h per ≥70% of nights were considered having “Good” adherence, while lower CPAP usage was considered as “Poor” adherence.

### Sleep study protocol

#### OSA screening study

Screening for the presence of OSA was performed using a type IV device (ApneaLink, ResMed, San Diego, CA, United States) that recorded hemoglobin saturation, heart rate and nasal airflow during sleep in a home setting. Subjects were instructed to set-up the device and keep regular sleep habits. Support in the form of a non-stop phone help-line was established and the devices were returned to investigators the next morning. Automatic scoring of respiratory events with a 4% desaturation threshold was performed, apneas defined as a ≥90% reduction in airflow for at least 10 s and hypopneas defined as a ≥30% reduction in airflow for at least 10 s together with hemoglobin desaturation of ≥4%. Patients with REI ≥15 were considered as being at high risk of moderate-to-severe OSA. For 13 patients the oxygen desaturation index (ODI) was used due to a poor airflow signal.

#### Diagnostic sleep study

Sleep recordings were performed using a type III device that recorded hemoglobin saturation, heart rate, nasal airflow, ECG, chest and abdominal respiratory efforts (Nox T3, Nox Medical, Reykjavik, Iceland) in a home setting. The recordings were evaluated by a board-certified sleep medicine physician according to AASM criteria (apnea defined by a ≥ 90% reduction in airflow for at least 10 s and hypopnea defined as a ≥ 30% reduction in airflow for at least 10 s together with ≥ 4% desaturation). Patients with moderate-to-severe OSA (REI ≥ 15) were recommended to initiate CPAP treatment.

### Statistical analysis

Statistical analysis was performed using Prizm 5 for Windows Software (GraphPad Software Inc., La Jolla, CA, United States). Differences in anthropometrical parameters between the patient groups were analyzed using a *T*-test and differences in frequencies were analyzed using a Chi-Square test. Data are presented as mean ± SEM, counts or proportions (%). Statistical significance was set to *p* < 0.05.

## Results

### OSA screening outcomes in type 2 diabetes patients

**Figure 1:**
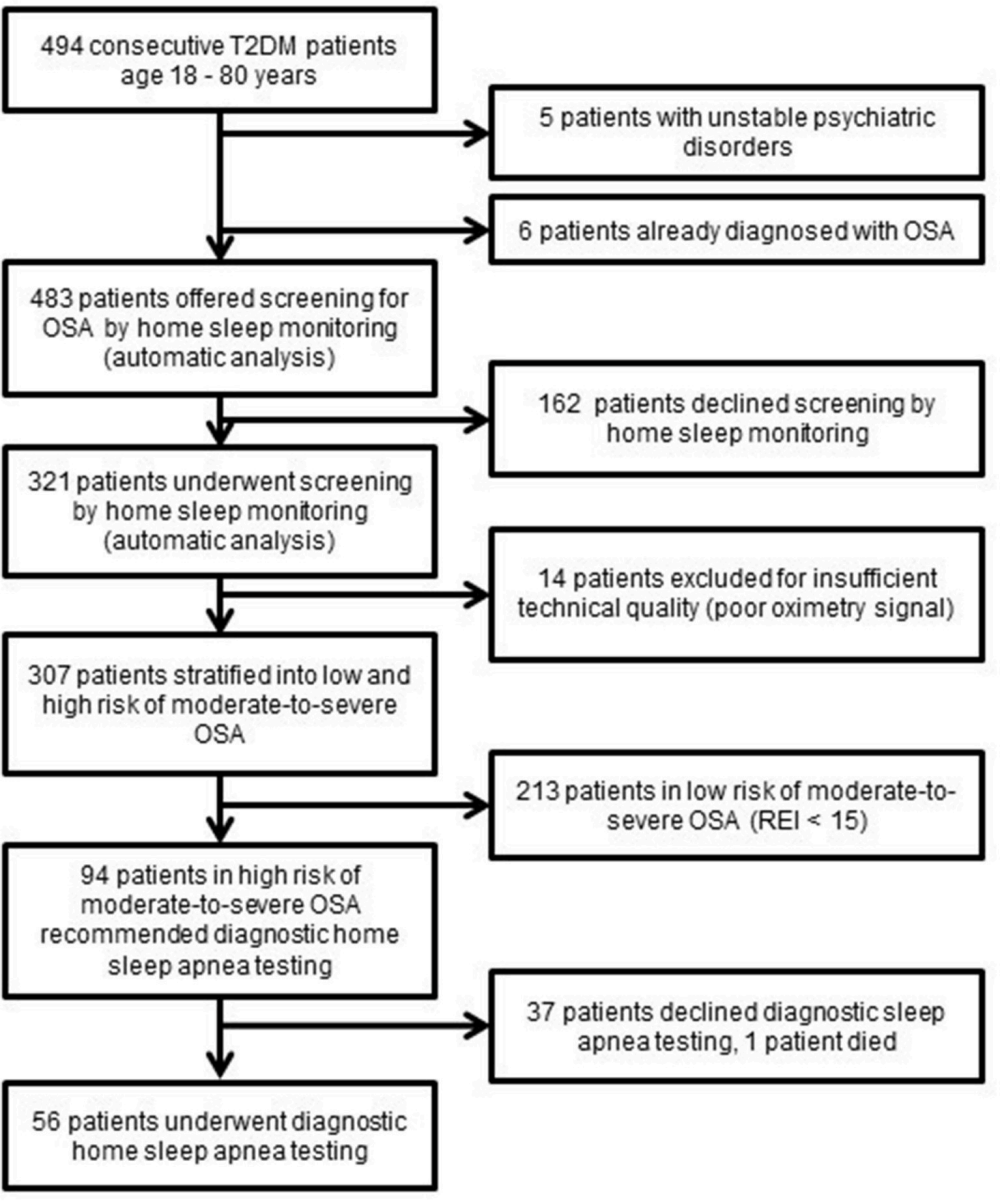
Flow diagram of Type 2 diabetes patients in the screening study T2D, Type 2 diabetes; OSA, Obstructive sleep apnea; REI, respiratory event index.

Out of 483 consecutive Type 2 diabetes patients who fulfilled the inclusion criteria, 321 patients consented to undergo OSA screening, resulting in 307 analyzed sleep recordings of an acceptable quality. Among successfully screened patients, 31% (63 men and 31 women) were identified as being in a high risk of moderate-to-severe OSA and thus invited for a diagnostic sleep study. However, such a sleep study was performed for only 60% of them due to the unwillingness of patients to further continue with the diagnostic process (Figure 1).

The Type 2 diabetes patients who accepted the diagnostic home sleep apnea testing (HSAT) were characterized by 42% higher REI (32.6 ± 2.4 vs. 22.9 ± 1.5, *p* < 0.05) and a 49% higher score in Epworth sleepiness scale (7.6 ± 0.6 vs. 5.1 ± 0.5, *p* < 0.05) than the Type 2 diabetes patients who declined HSAT. No differences in anthropometric and demographic parameters or associated comorbidities were observed (Table 1).

**Table 1:** Characteristics of type 2 diabetes patients screened for OSA by home sleep monitoring.

	**All**	**REI < 15**	**REI ≥ 15**	**REI** ≥ **15**	
				**declined HSAT**	**examined by HSAT**
Patients, *n* (%)	307 (100%)	213 (69%)	94 (31%)	37 (39%)	56 (60%)
Men, *n* (%)	177 (58%)	114 (54%)	63 (67%)	24 (65%)	38 (68%)
Age (years)	64.0 ± 0.5	63.7 ± 0.6	64.8 ± 1.0	65.1 ± 1.6	64.3 ± 1.3
BMI (kg/m^2^)	31.2 ± 0.3	30.4 ± 0.3	33.0 ± 0.6*	32.4 ± 0.8	33.4 ± 0.9
Hypertension, *n* (%)	255 (83%)	172 (81%)	83 (88%)	32 (86%)	46 (82%)
Dyslipidemia, *n* (%)	262 (85%)	182 (85%)	80 (85%)	32 (86%)	41 (73%)
CV disease, *n* (%)	46 (15%)	28 (13%)	18 (19%)	6 (16%)	10 (18%)
ESS, *n*	3.2 ± 0.2	5.8 ± 0.3	6.6 ± 0.4	5.1 ± 0.5	7.6 ± 0.6**
REI_screening study, *n*	12.5 ± 0.8	5.3 ± 0.3	28.7 ± 1.6	22.9 ± 1.5	32.6 ± 2.4**
REI_diagnostic study, *n*					37.7 ± 2.4
T90, %					23.6 ± 3.4

*BMI, body mass index; CV disease, cardiovascular disease defined as myocardial infarction, percutaneous coronary intervention or stroke, REI, respiratory event index; T90, percentage of total sleep time with oxygen saturation < 90%; HSAT, home sleep apnea testing; ESS, Epworth Sleepiness Scale*.

*Data represent mean ± SEM or proportions (%)*,

* *p < 0.5 for differences between REI < 15 group and REI ≥ 15.group (T-test, Chi-squared test)*,

** *p < 0.05 for differences between declined HSAT group and examined by HSAT group (T-test, Chi-squared test)*.

### CPAP acceptance and adherence in type 2 diabetes and sleep clinic patients

**Figure 2:**
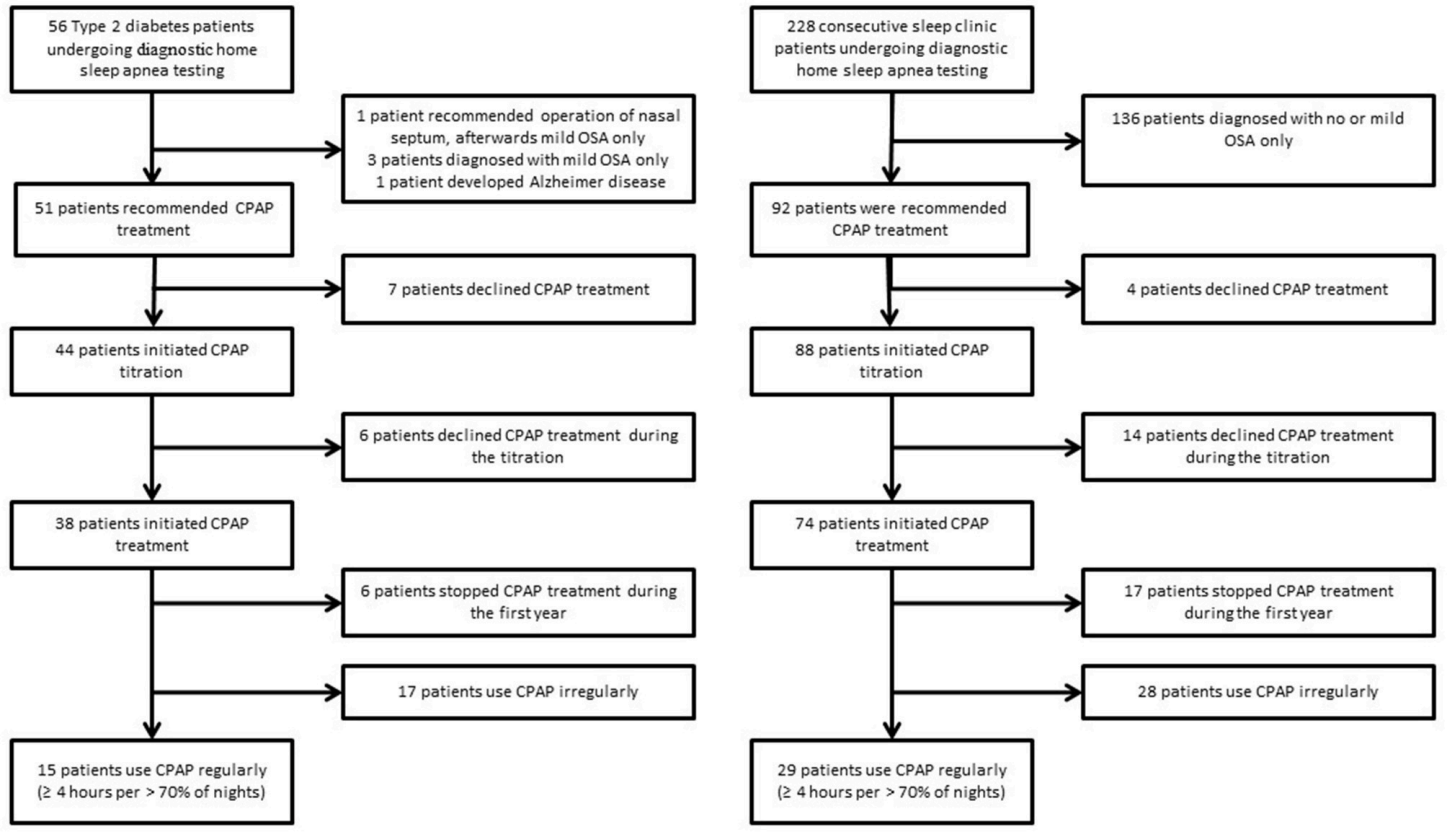
Flow diagram of Type 2 diabetes and sleep clinic patients undergoing diagnostic home sleep apnea testing.

Based on the results of home sleep apnea testing, 51 Type 2 diabetes patients were recommended to initiate CPAP treatment. However, 13 patients dropped out before or during the CPAP titration, resulting in a CPAP acceptance rate of 75% (38 treated patients). A similar acceptance rate of 80% (*p* > 0.05) was observed in sleep clinic patients−74 patients were treated out of 92 patients recommended for CPAP (Figure 2).

Type 2 diabetes patients who were recommended to initiate the CPAP treatment were, in comparison to sleep clinic patients who received same recommendation, older (64.4 ± 1.3 vs. 52.3 ± 1.4, *p* < 0.05), had a lower score in the Epworth sleepiness scale (7.7 ± 0.7 vs. 10.3 ± 0.7), and they were more frequently treated for hypertension and dyslipidemia. There were no significant differences in REI and time spent in saturation < 90% between the Type 2 diabetes and the sleep clinic patients who were recommended CPAP (Table 2).

**Table 2:** CPAP acceptance – comparison of type 2 diabetes and sleep clinic patients.

	**TYPE 2 DIABETES PATIENTS**			**SLEEP CLINIC PATIENTS**		
	**All recommended CPAP**	**Accepting CPAP**	**Not accepting CPAP**	**All recommended CPAP**	**Accepting CPAP**	**Not accepting CPAP**
Patients, *n* (%)	51 (100%)	38 (75%)	13 (25%)	92 (100%)	74 (80%)	18 (20%)
Men, *n* (%)	34 (67%)	23 (61%)	10 (77%)	75 (82%)	64 (86%)	11 (61%)*
Age (years)	64.4 ± 1.3	62.9 ± 1.6	68.8 ± 2.3	52.3 ± 1.4^#^	51.9 ± 1.4	53.7 ± 3.0
BMI (kg/m^2^)	33.8 ± 0.9	35.0 ± 1.1	30.2 ± 1.0*	34.3 ± 0.8	34.8 ± 0.8	32.1 ± 1.4
Hypertension, *n* (%)	46 (90%)	35 (92%)	11 (85%)	60 (65%)^#^	49 (66%)	11 (61%)
Dyslipidemia, *n* (%)	42 (82%)	32 (84%)	10 (77%)	41 (45%)^#^	35 (47%)	6 (33%)
CV disease, *n* (%)	10 (20%)	8 (21%)	2 (18%)	7 (8%)	5 (7%)	2 (11%)
ESS (score)	7.7 ± 0.7	7.6 ± 0.8	8.2 ± 1.5	10.3 ± 0.7^#^	10.7 ± 0.7	8.6 ± 1.0
REI, *n*	39.7 ± 2.5	40.8 ± 3.0	36.3 ± 3.7	41.9 ± 2.4	46.0 ± 2.4	24.8 ± 2.5*
T90, %	25.5 ± 3.6	31.3 ± 4.2	5.7 ± 2.3*	20.8 ± 3.0	24.7 ± 3.0	4.9 ± 1.8*

CPAP, positive airway pressure; CV disease, cardiovascular disease defined as myocardial infarction, percutaneous coronary intervention or stroke; ESS, Epworth Sleepiness Scale, REI, respiratory event index, T90, percentage of total sleep time with oxygen saturation < 90%.

Data represent mean ± SEM (standard error of the mean) or proportions (%)

* p < 0.05 for differences between patients accepting and not accepting CPAP within the group (Type 2 diabetes patients or sleep clinic patients) (T-test, Chi-Square test).

# *p < 0.05 for differences between Type 2 diabetes and Sleep clinic patients recommended CPAP (T-test, Chi-Square test)*.

Sleep clinic patients who accepted CPAP exhibited more severe OSA than patients not accepting CPAP (REI 46.0 ± 2.4 vs. 24.8 ± 2.5, *p* < 0.05). This difference was not observed in Type 2 diabetes patients accepting and not accepting CPAP (REI 40.8 ± 3.0 vs. 36.3 ± 3.7, *p* > 0.05), where some patients with lower REI determined by screening had already declined diagnostic home sleep apnea testing. Hypoxic exposure evaluated by time spent at saturation < 90% correlated with CPAP acceptance in both Type 2 diabetes and sleep clinic patients (Table 2).

The CPAP recordings obtained after a 1-year follow-up showed “good” adherence to CPAP treatment defined as CPAP usage ≥ 4 h in ≥ than 70% of nights in 15 out of 38 Type 2 diabetes patients and 29 out of 74 sleep clinic patients who initiated CPAP treatment resulting in a 39% adherence rate in both groups (Table 3). A comparison of patients with “good” adherence and “poor” adherence to CPAP revealed that sleep clinic patients with “good” adherence were characterized by a higher REI and a longer time spent in saturation < 90%, while no differences in sleep variables and anthropometric parameters were observed between Type 2 diabetes patients with good and poor adherence (Table 3).

**Table 3:** CPAP adherence 1 year after initiating treatment —comparison of Type 2 diabetes and sleep clinic patients.

	**TYPE 2 DIABETES PATIENTS**			**SLEEP CLINIC PATIENTS**		
	**All initiating CPAP**	**Good CPAP adherence**	**Poor CPAP adherence**	**All initiating CPAP**	**Good CPAP adherence**	**Poor CPAP adherence**
Patients, n (%)	38 (100%)	15 (39%)	23 (61%)	74 (100%)	29 (39%)	45 (61%)
Men, n (%)	23 (61%)	9 (60%)	14 (61%)	64 (86%)^#^	28 (97%)	36 (80%)*
Age (years)	62.9 ± 1.6	64.6 ± 2.2	61.8 ± 2.1	51.9 ± 1.4^#^	50.4 ± 2.4	52.9 ± 1.7
BMI (kg/m^2^)	35.0 ± 1.1	35.7 ± 1.7	34.6 ± 1.5	34.8 ± 0.8	35.3 ± 1.2	34.5 ± 1.2
Hypertension, n (%)	35 (92%)	12 (80%)	23 (100%)*	49 (66%)^#^	19 (66%)	30 (67%)
Dyslipidemia, n (%)	32 (84%)	13 (87%)	19 (83%)	35 (47%)^#^	18 (62%)	17 (38%)
CV disease, n (%)	8 (21%)	2 (13%)	6 (26%)	5 (7%)	4 (14%)	1 (2%)
ESS (score)	7.6 ± 0.8	5.9 ± 0.8	8.7 ± 1.1	10.7 ± 0.7^#^	10.6 ± 1.2	10.8 ± 0.8
REI, n	40.8 ± 3.0	44.0 ± 5.1	38.7 ± 3.8	46.0 ± 2.4	52.5 ± 4.1	41.8 ± 2.8*
T90, min	31.3 ± 4.2	38.1 ± 7.4	26.8 ± 4.9	24.7 ± 3.0	32.8 ± 5.1	19.5 ± 3.6*
T90 ≥ 10%, n (%)	29 (76%)	12 (80%)	17 (74%)	42 (57%)	21 (72%)	21 (47%)
CPAP ADU_all_, h	3.8 ± 0.4	5.8 ± 0.3	2.6 ± 0.3*	3.7 ± 0.3	6.1 ± 0.2	2.1 ± 0.3*
CPAP ADU_1year_, h	4.5 ± 0.3	5.8 ± 0.3	3.3 ± 0.2*	4.8 ± 0.2	6.1 ± 0.2	3.4 ± 0.3*

CPAP, continuous positive airway pressure; CV disease, cardiovascular disease defined as myocardial infarction, percutaneous coronary intervention or stroke; REI, respiratory event index; T90, sleep time with oxygen saturation < 90%, CPAP ADU_all_, CPAP average daily use of all patients accepting CPAP; CPAP ADU_1year_, CPAP average daily use of all patients using CPAP after 1 year; Good adherence to CPAP, average daily use of CPAP ≥ 4 h ≥ 70% of nights 1 year from initiating CPAP treatment;

Poor adherence to CPAP, average daily use of CPAP < 4 h ≥ 70% of nights 1 year from initiating CPAP treatment

Data represent mean ± SEM (standard error of the mean) or proportions (%)

* p < 0.05 for differences between patients with “good” and “poor” adherence to CPAP within the group (Type 2 diabetes patients or sleep clinic patients) (T-test, Chi-Square test)

# *p < 0.05 for differences between Type 2 diabetes and Sleep clinic patients initiating CPAP (T-test, Chi-Square test)*.

The proportion of patients with good adherence after 3 months and 1 year of using CPAP did not significantly differ (44.7 vs. 39.5% *p* > 0.05 and 40.5 vs. 39.2% *p* > 0.05 for Type 2 diabetes and sleep clinic patients, respectively). Some patients exhibited better adherence after 3 months than after 1 year of using CPAP and vice versa (Table 4). Adherence rates reported in this study range from 39 to 64% in Type 2 diabetes patients and from 39 to 68% in sleep clinic patients when various definitions of “good” adherence were used (Table 5).

**Table 4:** Differences in CPAP adherence 3 months and 1 year after initiating the CPAP treatment.

	**Type 2 diabetes patients**	**Sleep clinic patients**
All initiating CPAP	38 (100%)	74 (100%)
Good adherence in 3 months, *n* (%)	17 (44.7%)	30 (40.5%)
Good adherence in 1 year, *n* (%)	15 (39.5%)	29 (39.2%)
Good adherence in 3 months but not in 1 year, *n* (%)	4 (10.5%)	6 (8.1%)
Good adherence in 1 year but not in 3 months, *n* (%)	2 (5.3%)	5 (6.8%)

*Good adherence, average daily use of CPAP ≥ 4 h ≥ 70 of nights*.

**Table 5:** CPAP adherence in T2DM and sleep clinic patients 1 year after CPAP initiation.

	**Type 2 DM**	**Sleep clinic patients**
All patients initiating CPAP treatment after titration	38 (100%)	74 (100%)
Patients using CPAP ≥ 4 h/night ≥ 70% of nights, *n* (%)	15 (39%)	29 (39%)
Patients using CPAP ≥ 4 h/night on average, *n* (%)	18 (47%)	38 (51%)
CPAP ADU_all_, h (% of desired 7 h/night/patient)	3.77 (54%)	3.66 (52%)
CPAP ADU_1year_, h (%of desired 7 h/night/patient)	4.47 (64%)	4.75 (68%)

Type 2 DM, Type 2 diabetes mellitus;

CPAP ADU_all_, CPAP average daily use of all patients accepting CPAP;

*CPAP ADU_1year_, CPAP average daily use of all patients using CPAP after 1 year*.

## Discussion

The present study showed that systematic OSA screening identified 31% of consecutive Type 2 diabetes patients as having a high risk of moderate-to-severe OSA, nevertheless, only 16% of them demonstrated a measurable clinical benefit of such screening by accepting and adequately adhering to CPAP treatment. However, the present study did not observe differences in CPAP acceptance and adherence rates in patients with Type 2 diabetes when compared to a sleep clinic population.

Although such results seem disappointing, they are in line with a study reporting that 17% of patients with Type 2 diabetes in high-risk of moderate-to-severe OSA initiated CPAP treatment (25). Similarly, a study in heart failure patients showed that ~ 12% of patients in high risk of moderate-to-severe OSA participated in a full diagnostic process, accepted the CPAP treatment and subsequently exhibited good adherence to CPAP (24). The present study identified two key dropout points in the screening diagnostic process. First, when the patient is recommended to enter the screening program (dropout rate 34%). Second, after obtaining the screening results, when the patient is advised to continue with the diagnostic sleep study (dropout rate 39%). However, once diagnosed with moderate-to-severe OSA, CPAP acceptance and adherence rates in patients with Type 2 diabetes were not different compared to a sleep clinic population. Sleep clinic patients who accepted and better adhered adequately to CPAP exhibited more severe OSA then those who did not accept or did not adhere well to CPAP. Such difference was not observed in screened Type 2 diabetes patients probably due to the fact that those with lower REI and shorter time spent in saturation < 90% were more likely to drop out right after screening—the step that was skipped by sleep clinic patients.

Epidemiological studies have provided evidence that adequate CPAP use is crucial for improving health outcomes such as sleepiness (26), cardiovascular morbidity and mortality (27–29). Understanding that the goal of any screening process is to diagnose and treat a disease, it becomes important to define measurable outcomes of OSA screening in Type 2 diabetes patients by terms of CPAP acceptance and adherence and, if possible, to compare these values with published reports. However, such effort is hampered by the variability of CPAP adherence definitions in the literature and by the fact, that CPAP acceptance (the willingness to use CPAP after diagnosis) is often not reported at all. It needs to be emphasized that active promotion and encouragement to at least “try” the CPAP therapy increases the acceptance rate. However, it can be expected that more patients will subsequently drop out and thus decrease the adherence rate. Additionally, a universally recognized definition of “good” adherence is missing leading to variability in reported results. Calculated adherence rates might differ by up to 29% in one study, depending on the adherence definition [CPAP use ≥ 4 h on ≥ 70% nights (30) vs. CPAP use of ≥ 4 h/night on average (27) vs. adherence rate defined as a fraction of the CPAP use time out of a “desired” CPAP use of 7 h/night/patient (23)] as demonstrated in Table 5. Furthermore, adherence can be modified by other factors, such as including a run-in period in the study and excluding patients with lower than demanded CPAP use at the beginning of treatment/titration (17) or by socio-economic factors, e.g., a significant patient contribution to cover the cost of CPAP treatment (22). All these factors need to be acknowledged when interpreting the results of the present study, where “good” adherence was based on the CPAP use ≥ 4 h ≥ 70% of nights, the selection of which is supported by (1) the adoption of this criterion by Medicare in its coverage policy in the USA as well as (2) clinical conservatism and data robustness as this criterion provides the lowest adherence rate from the mentioned methods. Nevertheless, the question of an optimal adherence calculation and the minimum required CPAP usage time that brings health benefits remains a matter of controversy (26).

Barriers preventing better compliance with OSA treatment were shown to be of a complex nature ranging from patient characteristic, disease severity, technological factors, means of OSA diagnosis and CPAP delivery to psychological and cultural variables (31). We believe that improving CPAP adherence rates is likely to be a lengthy process that can possibly be helped by increasing patients awareness of OSA, its consequences and treatment options by physicians of different expertise, including general practitioners, cardiologists and endocrinologists either in person or indirectly such as by information leaflets in the waiting rooms. Furthermore, the patient attitude to OSA might be also improved by demonstrating a more friendly means for the treatment of OSA other than CPAP (i.e., oral appliances) to patients who find CPAP unacceptably.

The significance of this study is that it implemented systematic OSA screening in consecutive Type 2 diabetes patients and followed its outcome all the way through the diagnostic process to measure the effectiveness of the subsequent CPAP treatment. However, the limitations of the study should be noted. First, although a considerable number of 483 patients with Type 2 diabetes were included in the study, a severe dropout during the diagnostic process led to a relatively modest number of 38 patients who initiated the CPAP treatment and on whom the adherence to CPAP was followed. Second, some of the drop out during the diagnostic process might have been prevented if the first step of using a type IV device for screening was skipped and all patients directly underwent diagnostic monitoring using a type III device. Another way to lower the drop out during the diagnostic process might be to describe the alternative treatment option available to the patient—an oral appliance—in the event that clinically significant OSA is diagnosed. Oral appliances are not routinely available in the Czechia but might represent a more acceptable prospective way of treatment than CPAP for some of the patients and therefore could encourage some of those who dropped out to continue in the diagnostic process. Third, OSA was diagnosed using a type III device in a home setting and not by deploying the gold standard polysomnography. Nevertheless, such a diagnostic attitude is far more accessible than polysomnography and more likely reflects practice in everyday life. Fourth, the patients enrolled in the study, both Type 2 diabetes patients and sleep clinic patients, were diagnosed and treated at one sleep clinic, so CPAP acceptance and adherence can partly reflect the attitude of that particular clinic. On the other hand, the attitude of healthcare professionals to both groups of Type 2 diabetes patients and sleep clinic patients was by default the same as they were not aware of any grouping and therefore the comparison of CPAP acceptance and adherence between the groups is reliable. Finally, in the present study metabolic outcomes (e.g., glucose tolerance and HbA1c) and microvascular complications were not assessed, even though adequate CPAP adherence clearly represents a key factor determining its beneficial effects on glucose tolerance, insulin sensitivity (9, 10) and HbA1c—although outcomes of studies investigating the effect of CPAP on HbA1c are mixed (12, 32) Additionally, CPAP therapy might also ameliorate microvascular complications as slowing of the decline in glomerular filtration rate (33) and improvement in visual acuity (but not macular edema) (34) was reported after CPAP treatment in Type 2 diabetes. Despite the increasing evidence linking OSA with the development and progression of Type 2 diabetes, several aspects of CPAP treatment remain unclear and future studies are thus warranted, e.g., on the role of CPAP in prevention of microvascular complications. Furthermore, low CPAP acceptance rate (not only among Type 2 diabetes) warrants the search for alternative treatment options (35, 36).

In conclusion, every third to fourth patient with Type 2 diabetes suffers from clinically significant obstructive sleep apnea syndrome indicated for CPAP treatment whilst not being aware of his/her condition. Once diagnosed, their acceptance and adherence to CPAP did not differ from sleep clinic patients even though originally they did not actively seek medical advice regarding OSA symptoms. As almost half of the patients identified to be at high risk of OSA through screening were unwilling to undergo the subsequent diagnostic process, our study implies that factors preventing Type 2 diabetes from further medical evaluation should be targeted, probably through more extensive patient education combined with complex psychological approaches. A consideration should be also given to the possibility of conducting screening and diagnostic sleep studies in a single step using appropriate home sleep apnea testing devices.

## Author contributions

KW and AP recruited subjects with Type 2 diabetes, performed screening sleep studies, gathered data, participated in data analysis, participated in manuscript preparation. VD participated in subject recruitment, served as a study coordinator, helped in performing diagnostic sleep studies, participated in data interpretation, participated in manuscript drafting. MP scored sleep studies, was responsible for CPAP therapy in all subjects, recruited non-diabetic subjects from the sleep clinic, participated in data interpretation and manuscript drafting. JP designed and supervised the study, analyzed data and participated in interpretation of the results, edited the manuscript.

### Conflict of interest statement

The authors declare that the research was conducted in the absence of any commercial or financial relationships that could be construed as a potential conflict of interest.
